# Supplementary material for: PERMA-guided multi-topology graph neural networks for cross-cultural student well-being prediction
Source: PLoS One. 2025 Dec 16;20(12):e0338693. doi: 10.1371/journal.pone.0338693 (PMC12707682; doi:10.1371/journal.pone.0338693)
Supplement: S1 Appendix — (DOCX) [file pone.0338693.s001.docx]

S1 Appendix. Complete hyperparameter configuration.

This appendix provides comprehensive hyperparameter settings for the PERMA-GNN-Transformer model to ensure full reproducibility, addressing Reviewer #2’s request for complete documentation of optimizer parameters, weight initialization strategies, and scheduler details.

Table A1: Training Configuration and Optimization

| **Category** | **Parameter** | **Value** | **Notes** |
| --- | --- | --- | --- |
| **Optimizer(AdamW)** |  |  |  |
|  | Learning rate | 2x10^-4^ | Initial value |
|  | Beta1(β,) | 0.9 | First moment estimate |
|  | Beta2 (β,) | 0.999 | Second moment estimate |
|  | Epsilon(ε) | 1x10^-8^ | Numerical stability |
|  | Weight decay | 1x10^-5^ | L2 regularization |
| **Learning Rate Scheduler** |  |  |  |
|  | Type | Cosine Annealing | Smooth decay |
|  | **Warmup steps** | **500** | **Initial stabilization** |
|  | Minimum LR | 1x10^-6^ | Lower bound |
|  | Max epochs | 100 | Before early stopping |
| **Regularization** |  |  |  |
|  | Dropout rate | 0.1 | All layers |
|  | Gradient clipping | 1.0 | L2 norm threshold |
|  | Early stopping patience | 15 epochs | Validation MAE |
| **Data Splitting** |  |  |  |
|  | Batch size | 32 | Memory-performance balance |
|  | Train/ Val/ Test | 70%/20%/10% | Standard split |
| **Loss Weights** |  |  |  |
|  | Wellbeing loss (λ.) | 1.0 | Primary obiective |
|  | PERMAloss(λ,) | 0.8 | Auxiliary objective |
|  | Consistency loss (λ„) | 0.5 | Theoretical constraint |

Table A2: Model Architecture Parameters

| **Module** | **Parameter** | **Value** | **Rationale** |
| --- | --- | --- | --- |
| **PERMA Embedding** |  |  |  |
|  | Input dimension | 23(Lifestyle)/varies | Dataset-dependent |
|  | Embedding dimensionper PERMA | 128 | Each of 5 dimensions |
|  | Output dimension | 640(5x128) | Concatenated PERMAfeatures |
|  | Dropout | 0.1 | Regularization |
|  | Weight initialization | Xavier Uniform +psychology priors | Theory-driven (Section 3.2.2) |
| **Multi-Topology**  **GNN** |  |  |  |
|  | Number of graph types | 4 | Cosine/Euclidean/Style/PERMA |
|  | GCN layers pertopology | 3 | Depth-efficiency balance |
|  | Hidden dimension | 128 | Consistent with embedding |
|  | GAT attention heads | 8 | Multi-head attention |
|  | Head dimension | 32 | 256/8 |
|  | Edge weightthre shold | 0.3 | Weak connection filtering |
|  | Graph attention initial  weights | 0.25 (uniform) | Learnable dynamic parameters |
|  | Graph normalization | Symmetric | Numerical stability and  convergence |
|  | Weight initialization | Kaiming Normal | For ReLU activation |
| **PERMA-Aligned**  **Transformer** |  |  |  |
|  | Number of layers | 6 | Standard depth |
|  | Hidden dimension | 256 | 2x embedding dim |
|  | Number of attention  heads | 5 | **PERMA-aligned** (P/E/R/M/A) |
|  | Head dimension | 64 | 256/5(non-standard) |
|  | FFN dimension | 1024 | 4x hidden dim |
|  | Activation function | GELU | Transformer standard |
|  | Dropout (attention) | 0.1 | Regularization |
|  | Dropout (FFN) | 0.1 | Consistent configuration |
|  | Weight initialization | Linear(256>1)+  Sigmoid | Transformer standard |
| **Prediction Heads** |  |  |  |
|  | Wellbeing head | Linear(256>1)+  Sigmoid | Regression to [0,1] |
|  | PERMA dimensionshead | Linear(256→5)+  Sigmoid | Five-dimensional output |
|  | Weight initialization | Xavier Uniform | Output layer standard |

Computational Environment

Hardware: NVIDIA GeForce RTX 4090 (24GB VRAM), Intel Core i9-13900K, 128GB RAM

Software: PyTorch 2.1.0, PyTorch Geometric 2.4.0, CUDA 11.8

Training time: ~1.5 hours (Lifestyle dataset), ~30 minutes (International dataset)
